# The Cost of Loneliness: Assessing the Social Relationships of the Elderly via an Abbreviated Sociotype Questionnaire for inside and outside the Clinic

**DOI:** 10.3390/ijerph19031253

**Published:** 2022-01-23

**Authors:** Jorge Navarro, Miguel Cañete, Francisco J. Olivera, Marta Gil-Lacruz, Ana Gil-Lacruz, Pedro C. Marijuán

**Affiliations:** 1 Grupo de Decisión Multicriterio Zaragoza (GDMZ), Faculty of Economics, University of Zaragoza, 50005 Zaragoza, Spain; 2 Psychology and Sociology Department, Education Faculty, University of Zaragoza, 50009 Zaragoza, Spain; mcanete@unizar.es; 3 Department of Psychiatry, Hospital San Jorge (Psychogeriatric Program), 22004 Huesca, Spain; joliverap@aragon.es; 4 Psychology and Sociology Department, Health Science Faculty, University of Zaragoza, 50009 Zaragoza, Spain; mglacruz@unizar.es; 5 Business Department, School of Engineering and Architecture, University of Zaragoza, 50018 Zaragoza, Spain; anagil@unizar.es; 6 Independent Scholar Affiliated to Bioinformation Group, Instituto Aragonés de Ciencias de la Salud (IACS), 50009 Zaragoza, Spain; pcmarijuan.iacs@aragon.es

**Keywords:** loneliness, social relationships, sociotype questionnaire, elderly isolation risk, geriatric clinic

## Abstract

Gauging the social relationships of the elderly is a significant sociometric research subject and a deep biomedical concern—particularly after the COVID-19 pandemic. It is imperative for facultatives in primary care, for geriatric clinics, and for social care services. In this respect, this article explores the validity of an abbreviated version of the Sociotype Questionnaire (SOCQ), a tool previously developed by the authors for assessing the social relationships of the general population, now specifically addressed to the elderly population. The aim is to construct a 4-item dichotomous scale (SOCG-4) out of the 12 items of the original scale of the SOCQ, so that it can serve to discriminate among the patients in primary care and the geriatric clinic, helping the facultative to find those in need of social care or of psychosocial intervention. The population data have been obtained from a series of previous studies on social relationships in different segments of the elderly population (Ntotal = 915). The resulting abbreviated version of SOCG-4 was extracted by means of confirmatory factor analysis, with the congruence, validity, and relationship with the determinants as close to optimal. The significant correlations with SOCQ (0.82), UCLA (−0.55), Barthel (0.40), and other relevant tests are obtained. The test was also put to trial in a pilot study, being applied to 150 subjects via phone surveys, home visiting, and geriatric clinic—it becomes particularly useful for assessing the social relationships in geriatric clinic use. The 4-item Geriatric Sociotype scale (SOCG-4) appears as a valid measurement instrument for use in the clinic and in other social care instances.

## 1. Introduction

The metrics of social relationships of the elderly acquired, even before the COVID-19 pandemic, an increasing importance due to two major trends in contemporary societies. On the one hand, there is the general improvement of public health and the parallel demographic transition, which are causing a remarkable increase in the older population with inevitable tensions in health systems and in social care services. On the other hand, there are profound cultural changes related to lifestyle, communication technologies, increased individualism, diminished “social capital,” and weakened social-bonding structures, which remarkably diminished the preferred relational activities (face-to-face talk) and increased feelings of personal isolation. The impact on physical health and mental health of this rising social condition of isolation is difficult to estimate, as well as the burden it represents for social care and public health systems [1]. Some authors claim there is a genuine “epidemic of loneliness” to confront [2,3,4,5]. Without a doubt, this worsening situation makes it necessary to gather more kinds of quantitative data concerning the different contexts of analysis, intervention, and clinics among the elders. In this sense, developing a very brief metrics on social relationships (and particularly the lack of this), to allow the straightforward use for geriatric clinics, is the *leit motif* of this paper.

Since the COVID-19 pandemic, loneliness has become an even more significant concern [6]. The pandemic limited social contact and promoted teleworking and telematic relationships. The World Health Organization (WHO) already confirmed that the rate of loneliness has risen, and the prevalence of mood disorders has increased (above all, depression and anxiety, and also self-harm and suicide) and exacerbated pre-existing mental health conditions [6]. Economic analysis is throwing light on the multiple costs attributable to, or resulting from, the mental health burden accompanying the pandemic [7]. Loneliness and its associated mental disorders generate substantial costs for the individual, family, employer, and community, payments ranging from medical bills to sick leave days, to medication expenses, to home care services, to nursing homes.

Overall, the direct impact of loneliness on mental and physical health, as well as the social burden it causes, has already been highlighted by a number of researchers in fields as diverse as social economics [8,9,10,11,12], social psychology [13,14,15], mental health and psychiatry [2,16,17,18], and physiology [19,20,21], not to speak of medical practitioners directly involved in primary care, family care, and geriatric clinics [3,4,22,23,24,25]. One of the main concerns is that the increase in the aging population and growing loneliness are putting the sustainability of public healthcare systems at stake [26]. Indeed, the research on aging has come into a new era that marks an inflection point, with unique medical, economic, and societal implications [21]. Additionally, all these preexisting problems were magnified by the social impact of the COVID-19 pandemic.

Thereafter, gauging the level of the social interaction of the elderly population becomes an important research goal. In this respect, the Sociotype Questionnaire (SOCQ) already developed by this team [27] was addressed to evaluate the main dimensions of social interaction for the general population. Based on the “Social Brain Hypothesis” [28], it emphasized the adaptive nature of our social relationships and the general convergence on widely shared classes of social bonds and related face-to-face exchanges [29]. Although this Sociotype Questionnaire (counting only 12 items) was also applicable to the elderly, it was not sufficiently agile for use in the geriatric clinic, in primary care, or in social care screening.

The challenge, regarding a variety of geriatric interventions and in primary care, is the creation of an abbreviated questionnaire of preferably 4 items, reminiscent of the blocks of the Mini-Mental State Examination (MMSE) and other reduced questionnaires, specifically tailored to the necessities of the geriatric clinic, and covering the main dimensions of sociability. Thus, the objective of the present paper is to construct a 4-item dichotomous scale (SOCG-4) out of the 12 items of the original scale of the SOCQ, so that it can serve to discriminate among the geriatric patients in the clinic, helping the facultative to find those in need of social care or of psychosocial intervention. The reduced number of items, 4, and the dichotomic nature of the responses should configure an easy instrument to be kept handy on the desk of the geriatric or primary care facultative. In fact, there is a dearth of adequate indicators for systematic use in the geriatric clinic, so that the practitioner might easily detect the condition of loneliness [30,31]. Contributing to fill in that detection gap is an essential aspect of this research. Needless to say, the authors are fully aware that no single indicator of perceived or actual social isolation can fully assess the degree to which an individual is lacking social resources [31].

In addition to the obtention of the abbreviated questionnaire, we included a brief pilot study in Appendix A, where SOCG-4 was applied to a total of 150 subjects in 3 different modalities: via phone surveys, home visiting, and the geriatric clinic (50 subjects each). The SOCG-4 appears as a valid instrument, particularly useful in the geriatric clinic, and also in the other social care instances for which an easy discriminant tool may be needed.

## 2. Materials and Methods

The data of four previous studies were integrated to obtain the abbreviated Sociotype Geriatric Questionnaire. These studies were part of an official research project granted by the Spanish Ministry of Health (FIS PI12/01480): “The Sociotype: A new metrics on the structure and dynamics of social relationships.” The project incorporated different studies, some of them exclusively concerning the elderly population, which were addressed towards different research purposes, namely the assessment of social networking, communication modalities, institutionalization versus home dwelling, and gender differences in socialization. These studies shared basic concepts, methodologies, and survey platforms. All of them were quantitative, though initially a qualitative study was also undertaken [27].

### 2.1. Component Studies

#### 2.1.1. Study 1

The total sample for this study comprised of 1075 participants from the general population (18–95 years). Of these, 208 were 65 years or older and were incorporated into the SOCG-4 (N1). An online internet platform, “SurveyMonkey^®^”, was used for data gathering and the statistical support. The questions included a range of sociodemographic variables, the entire Sociotype Survey, and several complementary tests on loneliness, general health, and personality, including the Revised UCLA Loneliness Scale (RULS), the General Health Questionnaire (GHQ-12), and the Eysenck Personality Questionnaire-Revised (EPQ-R). This series of questionnaires was self-administered, with the availability of support staff when needed. As a result of the study, a 16-item Sociotype Questionnaire, SOCQ, was validated [27], which was counted using 4 main relational dimensions: family, friendships, acquaintances, and work/study when applicable. Otherwise, when the latter dimension was not applicable, i.e., for the elderly, SOCQ became a 12-item questionnaire.

#### 2.1.2. Study 2

This geriatric study involved a total of 275 participants (65 years or older). It was based on the same procedures and questionnaires as Study 1 (Sociotype Survey, UCLA, GHQ-12, and Eysenck). It addressed the elderly population living in their own homes. The goal of this study was to observe the quantitative differences of the elderly versus other age segments, regarding the averages of both social networking and daily talking time [29].

#### 2.1.3. Study 3

This geriatric study involved 283 participants (65 years or older), most of them enrolled in active and healthy aging activities organized by the Social Care Regional Institute (IASS), which oversees the active and healthy aging programs in the Spanish region of Aragon. Apart from the Sociotype Survey, this included several questionnaires that evaluated the specific characteristics of the elderly: the geriatric depression scale, the Pfeiffer questionnaire, and the Barthel ADL index. Its aim was to provide a comparison between age segments within the elderly population and to investigate the “minimum daily talking” (Navarro et al., in preparation).

#### 2.1.4. Study 4

This geriatric study compared physical and mental health status, and assessed the social relationships od elderly people in two different circumstances: institutionalized in residences versus living at home. It included the Sociotype Survey and some other questionnaires that evaluated in detail the mental and physical characteristics of the elderly: the geriatric depression scale, Pfeiffer questionnaire, Barthel ADL index, MiniMental, IAE cumulative index, and EuroQol-5D. The study population involved 200 individuals (70–97 years old), for which 100 individuals were institutionalized in residences (private and public) and 100 were living at home and attended daycare centers. All of them were interviewed by a psycho-geriatric facultative.

### 2.2. Participants

The total number of participants included in the present work is *N* = 915 (*N*1 = 208, *N*2 = 275, *N*3 = 283, and *N*4 = 200, respectively, belonging to studies 1, 2, 3, and 4. The inclusion criteria were being 65 years or older (70 years old in Study 4), being able to read and write Spanish, and not suffering from severe psychosis or not being diagnosed with severe cognitive impairment. The different samples were mainly composed of Caucasian adults, between 65–97 years old (mean = 76.95; SD = 8.02). All of them were Spanish (with diverse regional backgrounds, but mostly from Aragon); 70.6% were women and 29.4% were men, between the ages of 65–97 years (mean = 76.95; SD = 8.02); 40.7% of them had a partner or were married and 44.7% were widows/widowers; 38.6% lived alone; 33.9% lived with their partner; 11.5% lived in residences; and 16.5% accounted for other conditions. The main sociodemographic characteristics of all the participants are shown in Table 1.

### 2.3. Procedure and Ethics

The procedures for Study 1 and Study 2 took approximately thirty minutes. Each participant was given information about the study, which included the aims of the project, the advantages/disadvantages of participation, a letter of informed consent, and an assurance of anonymity (in line with the Spanish Organic Law 15/99 on the Protection of Personal Data, and Law 41/02 on Patient Autonomy). A research psychologist or a hospital nurse were on hand to give support where required. The survey of Study 3 implied a similar procedure, though the response time was shorter, in the order of twenty minutes. The methodologies and questionnaires of these studies were previously approved by the Ethical Committee of Aragón (CEICA), Spain (Act: CP13/2014). In the case of Study 4, the response time was in the order of fifty minutes, and its approval was granted by the Ethical Committee of the Hospital Complex of Navarre (Act: 18 January 2017).

### 2.4. Statistical Procedure

The statistical analysis was conducted with SPSS software (IBM SPSS Statistics for Windows, Version 24.0, IBM, Armonk, New York, NY, USA). The description of the population characteristics was by means of numbers and percentages for the categorical variables, whilst the mean and standard deviations (SDs) were employed for the quantitative variables.

The objective of the statistical analysis was to construct a 4-item dichotomous scale (SOCG-4) out of the 12 items of the original scale of the SOCQ. To achieve that goal, three phases were established:

- First phase: selection process of the most representative item within each of the three dimensions of the SOCQ. To accomplish this, the weights from the exploratory factor analysis by the Principal Component method were taken as references (previously making sure that these factors for elderly people were no different from those obtained for the general population).
- Second phase: for the selection of a 4th item, we searched among all the other discarded items (removing those previously selected), and we selected that which obtained the highest correlation with the GDS, Barthel, Pfeiffer, EPQ, UCLA, GHQ12, MMSE, EuroQol-5D, and Goldberg scales (taking into account that each test only applied to some determined population).
- Third phase: once we selected the 4 items, they were dichotomized assigning the value of 1 to the answers of the upper categories of the SOCQ and the value 0 to the remaining categories. Then, the validity of this new, “binary” scale was confirmed.

In order to confirm the validity of the procedure, the correlations of the resulting scale (SOCG-4) were recalculated with all the tests referred to in the second phase, corroborating that these correlations were significant, and that the questionnaire had predictive validity.

## 3. Results

Given the methodology proposed, which in its first phase involved selecting the weights from the exploratory factor analysis by the principal component method, it was necessary to previously inspect the weights and h^2^ values corresponding to the SOCQ questions for the aging population versus the general population. This could be conducted via the data of Study 1, where the values for general population were obtained, but a fraction of 208 individuals corresponded to the aging population.

### 3.1. SOCQ Differential Results in Study 1: The Aging Population versus General Population

By searching for the reliability of the SOCQ questions among the aging population, we can observe, in Table 2, that the results obtained also justify the 12 questions for the aging population; however, we observe an interesting difference regarding the weights and the h^2^ values of most of those questions with respect to general population.

As Table 2 clearly shows that the new weights upon which the present study is based are different from those in Table 3 below, that reproduces the values corresponding to the general population [27]. Following the methodology proposed, the items selected in Phase 1 were different in at least two cases (the 2nd and 3rd, questions, and probably the 4th one too—see later). Subsequently, the differences found between the general population and the elderly become the determinants for the extraction of the abbreviated scale we are looking for.

### 3.2. Construction of the Abbreviated Geriatric Sociotype Scale (SOCG-4)

Phase 1: following the factorial analysis based on the principal components method, we obtained a Kaiser–Meyer–Olkin index of the sampling adequacy of 0.845, with the Bartlett sphericity test χ266 = 5448.963 and *p* < 0.001. These values indicate that the factorial model is good and that it is therefore appropriate to carry out this technique based on the correlations found between the items. We used the Varimax orthogonal rotation procedure since the SOCQ was composed of three independent dimensions [27].

After conducting the factorial analysis, we found a 67.44% of total variance explained by the 3 factors, the most important being the friends factor with an explained variance of 38.59%, followed by the family factor with 17.73%, and finally the acquaintances factor with 11.12%. Since our interest was that the three dimensions of the SOCQ were represented in the final scale, we incorporated an item from each of the three dimensions—the one with the highest factorial weight (see Table 2). The selected items were: the 1st item in the family dimension (w_1_ = 0.873), the 5th item in the friends dimension (w_2_ = 0.902), and the 10th item in the acquaintance dimension (w_3_ = 0.765).

Phase 2: we discarded for this phase the three items already selected. Additionally, for the selection of the fourth item of the simplified scale, we observed the correlations with the remaining scales used (GDS, Barthel, Pfeiffer, EPQ, UCLA, GHQ12, MMSE, EuroQol-5D, and Goldberg), considering the different populations associated. Of the 9 remaining items of the SOCQ, the one that had a greater correlation with these scales was the 8th item, belonging to the friends dimension. Therefore, this was chosen as the 4th item of the abbreviated scale. The final result was, then, the following scale:

*I talk and relate with my family*;
*I talk and relate with my friends*;
*(R)It is difficult for me to make conversation with people I do not know*;
*I have fun and laugh with my friends*.

Phase 3: the last step of the procedure was to dichotomize the four items and check the psychometric properties of the dichotomized final scale. The three highest values of response in each of these 4 items were assigned the value of 1, and the three lower ones the value of 0. The reliability coefficient (Cronbach’s α) for the final scale was 0.57 (*N* = 915) and the correlations with the remaining tests are shown in Table 4.

As Table 4 shows, the correlation between both sociotype questionnaires was very high. In addition, the abbreviated scale had a high positive correlation with the extraversion subscale of the Eysenck EPQ-R, indicating that the higher the score in SOCG-4, the greater the extraversion; the relationship with the revised UCLA loneliness scale was also relevant, showing a strong negative correlation. The whole correlations of Table 4 will be discussed later.

Additionally, in Study 3, about the elderly population living at home versus those institutionalized in residences, we took the values corresponding to the 4 items of the abbreviated questionnaire and dichotomized them, obtaining the results below (Table 5):

## 4. Discussion

We obtained the new version of the target questionnaire of 4 items with 2 response options, by extracting 4 among the 12 original items and dichotomizing the original 6 response options from the SOCQ. This abbreviated SOCG-4 questionnaire for the geriatric population was put to trial in a pilot study, shown below in Appendix A.

In the sociodemographic data of our studies, men appear as underrepresented in the sample composition, as is common in many studies of the elderly population [32]. Most participants were widows/widowers (44.7%), and 38.6% lived alone—these two conditions become potential factors of perceived loneliness, specially the former. This was observed in the sociotype study for social relationships in the geriatric population [29], in which the widow/widower condition approximately cuts in half the average conversation time of the surviving partner. We can consider that living alone and being a widow represent two widespread sociodemographic features of the elderly population that, together with the low-income level (35.8% of our sample was below the average pension) and poor health, may have an important effect on social relationships. The geriatric facultative should carefully ponder these conditions as genuine risk factors of perceived loneliness, in addition to specialized questionnaires, such as the abbreviated ones observed herein.

The results presented in Table 2 and Table 3 are evidence that the discriminant quality of the 12-item SOCQ in the general population may be mirrored in the elderly population as well. However, slight differences appear between both tables, which would be more significant if those differences could be extracted by specifically comparing the disjointed age groups (the general population of Study 1 contained close to 20% of the elderly population). The weights obtained for the different questions in the sociotype’s three dimensions, show clear differences that precisely guided the selection procedure in the three phases already mentioned.

Regarding the four questions finally selected, they may be interpreted attending to their sociological scope. Question 1, “I talk and relate with my family”, belongs to the emotional loneliness component, related to the presence or absence of intimate relationships with a partner or a best friend [30]. We may consider that it partially overlaps with Question 2, “I talk and relate with my friends”, when strong bonds of friendship exist. However, in general, the emotional attachment to friends is lower than it is to partners—see the dramatic change in the talking time of the widows/widowers already mentioned [29]. The social loneliness aspect would then be covered by Question 2, and also by Question 3 (in reverse): “It is difficult for me to make conversation with people I do not know.” This point on weak or nil relatedness is important. For the capability to uphold the mental effort that conversation with unknown parties usually implies (and the extra reward often obtained), seems to be a significant indicator of mental health and the personal potential for active aging and longevity. Remarkably, this was found by Julianne Holt-Lundstat in a series of relevant population studies [3,4]. Rather surprisingly (not so from the sociotype point of view), maintaining social relationships with unrelated individuals lists as the first factor for predicting longevity, immediately followed by close family relationships. All the usual predictors and conventional advice, such as exercising, quit smoking and drinking, diet, flu vaccination, and going to the doctor, appear later in the list. Clearly, complete social engagement, a rich sociotype, is a prerequisite for physical and mental health, especially for elderly persons. Therefore, it is important to rely on a simplified but valid instrument to assess this.

Concerning Question 4, “I have fun and laugh with my friends”, the appearance of laughter among the selected questions may look odd. In fact, none of the existing questionnaires on the related topics (e.g., UCLA loneliness scale, MSPSS, SNI, Duke, SELSA, MOS, SSB, Zimet, and de Jong) include laughter. Is it an anomalous presence? Far from that, the research by some of the present authors [33,34,35] and by many others [36,37,38,39] indicate that laughter is closely related to the development of sociality and particularly to the formation and maintenance of social bonds. Furthermore, according to the population studies of Hassan and Hassan [38], the frequency of laughter is an excellent predictor of good physical health and mental health, as can be confirmed in medical stories themselves. In this research, once the three questions with the highest scores were selected, this fourth question centered on laughter obtained the best scores in its correlation with the other health questionnaires.

Beyond the excellent correlation with the SOCQ (0.82), in Table 4, the abbreviated dichotomous scale of SOCG-4 correlates very well with UCLA’s detection of feelings of loneliness and social exclusion (−0.55) and with the GDS metrics of depression (−0.105), as well as with the extraversion subscale of Eysenck (0.58). It means that the abbreviated questionnaire gauges the feelings of loneliness quite soundly and captures very well the personal differences in extroversion. Concerning the acceptable value of the correlation with GHQ12 (−0.36), it indicates that poor health and psychological distress have an impact on social engagement, and vice versa. This result was not unexpected, for loneliness has already been proved to be a most important risk factor for both the physical health and mental health of the elderly [2,22,40]. The relationship with the scale of physical activities of daily life (Barthel scale) was also positive (0.30), with a better performance for people with high scores in autonomy. Finally, another remarkable correlation appears with the EuroQol-5D scores, also in the vicinity of 0.5 (−0.45 for distress in the quality of life and 0.47 for the global health index, respectively). All these significant correlations indicate the potential usefulness and validity of SOCG-4 as an auxiliary tool concerning the screening of the old population in geriatric clinics and primary care, with the obvious advantages of its very easy use and friendly questioning, always maintaining the necessary cautions when relying on a very simple, dichotomous scale. As an indicative example of its use, in Appendix A below, we describe the first fieldwork experience using this questionnaire in three different contexts: in phone attention, in home visiting, and in the clinic.

About the results in Table 5, obtaining SOCG-4 values for the elderly population living at home vs. those institutionalized in residences, the differences are very significant, indicating that the questionnaire is valid to discriminate between the two groups (Student’s *t*-test and Cohen’s d clearly indicate this). The standard error corresponds to the average misclassification error when only looking at the values of the SOCG-4 questionnaire. Moreover, in the population of Study 3, the correlation between SOCG-4 and SOCQ reaches 0.89, higher than the general 0.82 that appears in Table 4 for the combined population of all the studies.

Finally, regarding the relationship between the abbreviated sociotype questionnaire and other relatively similar tests, such as perceived social support [41], social-emotional loneliness [30], or the abbreviated UCLA [42], despite circumstantial resemblances, there is an important difference with them. The latter tests have an implicit sense of dependence, vulnerability, counting on alien support for covering personal needs, or even directly assuming a depressed state, while the sociotype refers to unmediated relationships, spontaneous talking, and a sense of empowerment while the subject carries her/his relationships autonomously. The subjects responded far more easily and with a better mood to the SOCQ and SOCG-4 questions, in particular to the question on laughter. Additionally, this user-friendliness was authenticated by social workers already using institutionally the SOCQ questionnaire (in comparison with UCLA), and by the pilot fieldwork use of SOCG-4, described in Appendix A. The results obtained in the fieldwork and the comments by the interviewers made clear the simplicity, usefulness and discriminating capability of the new questionnaire.

Thus, by comparing the performance of the new questionnaire with the other abbreviated tests, we may speculate that, in general, the degree of usefulness of say “positive” versus “negative” constructs would depend on the level of autonomy of the subject. For instance, in the case of relatively young elderly individuals, the positive construct would fare better as a tool for distinction, while for the oldest segments (or “fourth age”) there would be an added capability to distinguish by the negative constructs. In any case, the correlations obtained for SOCG-4 with the other positive/negative tests in Table 4 look really promising, since it is a simplified, dichotomous test of only four questions. It is relevant that in the preliminary use of the new questionnaire, it has shown sufficient sensibility to establish the distinctions between the different populations in the three contexts.

## 5. Conclusions

The most important protection for the elderly, counting with a modicum of social relationships, is often left out of scope in current medical care, highly segmented and focused on organ failures and age-related diseases [21]. By paying special attention to social isolation and perceived loneliness, the primary care doctor or the geriatric facultative will be, in fact, monitoring the state of the most important risk-factor for the patient’s health.

Thereafter, prescribing “socialization pills”, via the coordination with municipal or regional day centers for the isolated elders, may raise the quality of life and in some cases prevent anti-depressive medication and the harmful syndrome of polypharmacy in the elderly individuals [21]. This implies the development of integrative management plans for general health, and the necessary interrelationship between medical care and social care institutions. It is not only the sustainability of health care systems that is at stake, but the betterment of daily lives for a growing population of increasingly isolated elders.

In this context, what the sociotype initiative means, and particularly SOCG-4, is the promotion of an easy instrument to be kept handy on the desk of the primary care and the geriatric clinic—acting as a reminder of the main health risk-factor of the old person standing in front of the doctor. We may insist on the importance of this new abbreviated metric, given the increase in loneliness after the COVID-19 pandemic.

Some of the SOCG-4 features may deserve ample consideration, such as simplicity, conviviality, user-friendliness, and inclusion of laughter. From a practical point of view, we explain in Appendix A how this fared in a phone survey, in home visiting, and in the clinic arenas. However, irrespective of the concrete performances of SOCG-4 and its basic limitations, it should stimulate discussion to contribute to a change of mentality in today’s care system, refocusing on the socialization needs not so well covered amidst the frequent administrative and technological shifts. Rapidly aging societies across the world demand new orientations, new intervention strategies, and new screening instruments for the gerosciences. The post-pandemic society desperately needs this type of approaches.

## Figures and Tables

**Table 1:** Sociodemographic data.

		Number	% Population
Gender	Women	646	70.6%
	Men	269	29.4%
	Total	915	100.0%
Age segments	From 65 to 75	333	38.5%
	From 76 to 85	406	46.9%
	More than 85	126	14.6%
	Total	865	94.5%
Stable relationships	Married/with partner	372	40.7%
	Single	102	11.1%
	Separate/divorced	32	3.5%
	Widow/widower	409	44.7%
	Total	915	100.0%
Connivence	Alone	353	38.6%
	Partner	310	33.9%
	Partner and offspring	50	5.5%
	Other family	53	5.8%
	Friends	2	0.2%
	Residence	105	11.5%
	Other	42	4.6%
	Total	915	100.0%
Education	Illiterate	28	3.1%
	No studies	372	40.7%
	Primary	324	35.4%
	High school	99	10.8%
	University	79	8.6%
	Other	13	1.4%
	Total	915	100.0%
Income	No income	35	7.3%
	Minimal pension	137	28.5%
	Average pension	232	48.3%
	High pension	61	12.7%
	Maximal pension	15	3.1%
	Total	480	52.5%

**Table 2:** Psychometric features of the SOCQ * (for the aging population in Study 1, *N*1 = 208).

SOCQ *	Mn	SD	DI	h^2^	w_1_	w_2_	w_3_
**Family**							
1. I speak and relate with my family	4.35	1.13	0.48	0.79	0.87	0.07	0.15
2. My family is important to me	4.73	0.85	0.40	0.66	0.80	0.03	0.11
3. The family members care about me	4.40	1.18	0.46	0.78	0.87	0.08	0.11
4. I have fun and laugh with my family	3.63	1.40	0.48	0.62	0.72	0.32	0.04
**Friends**							
5. I speak and relate with my friends	3.21	1.71	0.69	0.85	0.10	0.90	0.17
6. I have friends to tell and share problems	2.89	1.85	0.65	0.82	0.05	0.88	0.21
7. I consider it important to maintain relationships with friends	3.78	1.62	0.63	0.76	0.11	0.85	0.16
8. I have fun and laugh with my friends	3.02	1.64	0.69	0.77	0.23	0.83	0.18
**Acquaintances**							
9. I speak and relate comfortably with acquaintances	3.83	1.27	0.57	0.54	0.27	0.29	0.62
10. It is difficult for me to make conversation with people I do not know (r)	3.08	1.59	0.32	0.59	0.04	0.05	0.76
11. It is easy for me to win support from acquaintances	2.57	1.58	0.29	0.31	0.02	0.19	0.53
12. Relations with my acquaintances are forced (r)	3.76	1.27	0.41	0.60	0.14	0.12	0.75
% of variance (real-data)					17.73	38.59	11.12

SOCQ * Exploratory Factor Analysis; Mn = mean. SD = standard deviation; w_1_, w_2_ and w_3_ = weights on the first-order factors; h^2^ = communality; and r = reverse score. For all items from 1 to 12, the range of values is 0–5.

**Table 3:** Psychometric features of the SOCQ (for the general population, *N* = 1075).

General SOCQ	Mn	SD	h^2^	w_1_	w_2_	w_3_
**Family**						
1. I speak and relate with my family	4.39	0.97	0.81	−0.16	0.94	0.02
2. My family is important to me	4.74	0.76	0.83	−0.12	0.91	0.10
3. The family members care about me	4.49	1.00	0.64	−0.04	0.81	−0.01
4. I have fun and laugh with my family	3.65	1.20	0.43	0.26	0.55	−0.12
**Friends**						
5. I speak and relate with my friends	3.44	1.48	0.81	0.89	−0.06	0.09
6. I have friends to tell and share problems	3.45	1.65	0.83	0.92	−0.07	0.06
7. I consider it important to maintain relationships with friends	4.14	1.39	0.81	0.90	−0.01	0.01
8. I have fun and laugh with my friends	3.59	1.41	0.68	0.82	0.09	−0.11
**Acquaintances**						
9. I speak and relate comfortably with acquaintances	3.61	1.19	0.47	0.06	0.12	0.61
10. It is difficult for me to make conversation with people I do not know (r)	3.19	1.33	0.34	−0.01	−0.08	0.61
11. It is easy for me to win support from acquaintances	2.29	1.48	0.24	0.08	−0.09	0.52
12. Relations with my acquaintances are forced (r)	3.53	1.05	0.42	−0.02	0.05	0.63
% of variance (real-data)				38.70	18.80	13.90

SOCQ Exploratory Factor Analysis; Mn = mean; SD = standard deviation; w_1_, w_2_ and w_3_ = weights on the first-order factors; h^2^ = communality; and r = reverse score. For all items from 1 to 12, the range of values is 0–5.

**Table 4:** Correlations of the dichotomous simplified SOCG-4 Scale.

		Range of Values	Mn	SD	SOCG-4 Correlation	*N*
**SOCG-4**		0–4	2.98	1.12	1.00	915
**SOCQ**		0–60	42.65	11.31	0.82 **	915
**GDS**		0–14	2.90	3.07	−0.10 **	469
**PFEIFFER**		0–1	0.08	0.18	−0.22 **	473
**BARTHEL**		5–105	95.48	19.31	0.40 **	469
**GHQ12**		5–36	13.71	6.20	−0.36 **	420
**UCLA**		20–69	35.35	10.85	−0.55 **	418
**MMSE**		0.20–1	0.65	0.17	−0.020	200
**GOLDBERG**		1–2	1.72	0.39	0.05	200
**EuroQol5D**	**Total**	1–2.83	1.60	0.44	−0.45 **	200
	**Health**	1–10	6.46	2.02	0.47	199
**EPQ-R**	**E**	0–19	9.72	4.75	0.58 **	409
	**N**	0–23	11.08	5.23	−0.14 **	408
	***p***	0–16	4.83	2.90	−0.04	408

** The correlation is significant at the 0.01 level (bilateral).

**Table 5:** Comparative SOG-4 in the Study 3 population (residence vs. home dwelling).

	Provenance	*N*	Mn	Sd	Standard Error
SOCG-4	Home dwelling	100	3.00	1.19	0.12
	Residence	100	1.99	1.37	0.14

Student’s *t*-test = 5.56; *p* < 0.001; effect size: Cohen’s d = 0.787; and range of values 0–4.

**Figure A1:**
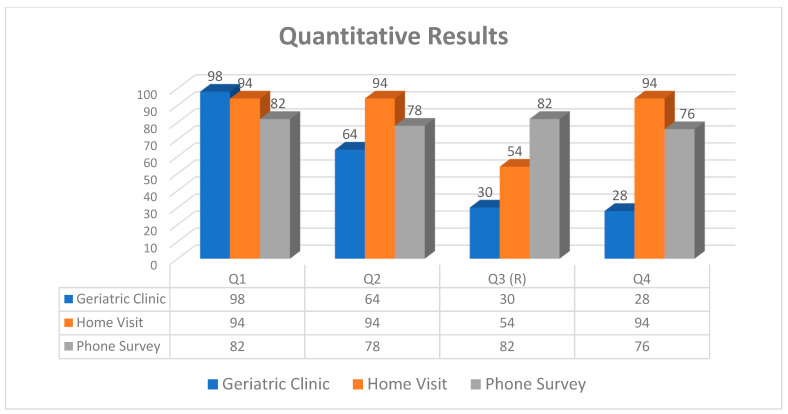
Quantitative results of SOCG-4 in three different contexts: geriatric clinic, home visit, and phone survey.

## Data Availability

The data presented in this study are available on request from the corresponding author.

## Appendix A. Use of SOCG-4

SOCG-4 was put to use in three different contexts: phone survey, home visiting, and psychogeriatric clinic. The two former cases were in charge of specialized employees of a social work company, while the latter case was in charge of a psycho-geriatric facultative, one of the authors of this paper, at San Jorge Hospital (Huesca, Spain). A total of 150 subjects were administered the abbreviated scale: 50 subjects in each modality. The social workers involved in the interviews were naïve regarding SOQG-4, and they did not receive any previous directions on how to administer the questionnaire and how the questions themselves should be asked or complemented. A casual mode, embedded within the usual procedure, was the only suggestion. Records were minimized or suppressed, and just the responses to the questions were consigned (no names, no indicators, no sociodemographic data, and no other tests). Apart from consigning the responses, only a qualitative impression was obtained. In the clinic case, age and sex were also annotated by the facultative.

### Appendix A.1. Comments from Interviewers

*Phone use.* Questions were easy to formulate by the specialized personnel (obviously in all cases the questions had to be changed from first person to second person). Most parties responded positively to the first question about family, and also to Question 2 about friends, but not without some hesitation or shame when their response was, or should have been, negative. Being asked about friendly relationships without face-to-face report made this question more difficult. In general, the response to this question was closely related to Question 4 on laughter. Nevertheless, administering the test was useful to provide a rough picture of the social relationships of those interviewed and, frequently, it also facilitated engaging in further phone conversations by the interviewer.

*Home use*. In this case, the difference between “preventive” users of the home-visiting service and those involved in the “dependence” official program was evident. The latter suffered from pathologies or physical/mental handicaps that restricted their capability for autonomous deambulation and outdoor activities. The positive response to the relationship with family was stronger in these dependent parties, while they easily acknowledged their missing social relationships—but not in the case of the preventive users of the service. An interesting gender difference was noted by the interviewer in charge of the survey; regarding Question 3, difficulty with strangers, men were responding more directly and positively than women. At stake is whether it was more a matter of maintaining a conventional feminine self-image or a genuine behavioral preference.

*Clinic use*. It was a very positive experience from the beginning. Older people who came to a consultation for problems of depression, cognitive impairment, or anxiety were pleasantly surprised when asked about family and friends. However, particularly regarding Question 1, about family, some of them were trying to please the facultative. For example, those who lived in a residence often commented that they received frequent visits from relatives and this was not really the case—the residence companions told the facultative about that. Those patients did not like to cast a negative image on themselves regarding their family bonds. In the other questions, that kind of obstacle was not observed and there was less trouble to show the degrees of personal isolation. In Question 3, for instance (difficulty with strangers), they answered that this was indeed the case. Older people had more difficulty in “opening up” to unknown parties, perhaps due to more fear and insecurity. In Question 4 (laughter), having fun and laughing with friends was complicated for the adults with depression or dementia. This question often caused them certain sadness when they found out that this was not their case. The affective state also influenced the responses. The depressed and sad individuals tended to respond that they did not talk or relate to family and friends, while their companions often commented that it was more “a feeling” than a reality. Overall, the test worked very well and most patients had a positive sensation of increased attention and personal care by the facultative.

### Appendix A.2. Quantitative Results

The results in Table 1, expressed in %, represent the positive responses to the different questions, with the proportion of “1s” obtained. In Question 3, the response is reversed (counting then the “0es”). Among the three contexts, the geriatric clinic use of the test appeared as the most sensitive, by far. With respect to Q1, however, the reluctance to accept poor family relationships was remarkable in all instances. Thus, a good suggestion could be the use of a complementary question just after the positive response (for instance, “Have you talked with them this week or last week?” or, “When was the last time you talked with them?”).

Regarding the discrimination of patients with a relative risk of isolation, there were noticeable differences between the outcomes of the different contexts. The clinic use was producing risk signals in 31 cases out of 50 (counting two or more “0es”), while in the other 2 contexts the warning signals were, respectively, 11 (phone) and 5 (home visiting). The disparaging results in Questions 3 and 4, in between the three contexts, might be partially explained by the relatively worsened conditions of geriatric patients and home visiting users. In any case, this was only a preliminary use of the test; rigorous fieldwork should be developed alongside future research by the authors.
